# The role of mitochondrial/metabolic axis in development of tamoxifen resistance in breast cancer

**DOI:** 10.1007/s13577-023-00977-5

**Published:** 2023-08-30

**Authors:** Hany N. Azzam, Marwa O. El-Derany, Sara A. Wahdan, Reham M. Faheim, Gouda K. Helal, Ebtehal El-Demerdash

**Affiliations:** 1 Department of Pharmacology and Toxicology, Faculty of Pharmacy, Heliopolis University, Cairo, Egypt; 2 Department of Biochemistry, Faculty of Pharmacy, Ain Shams University, Cairo, Egypt; 3 Department of Pharmacology and Toxicology, Faculty of Pharmacy, Ain Shams University, Cairo, Egypt; 4 Department of Clinical Oncology and Nuclear Medicine, Faculty of Medicine, Ain Shams University, Cairo, Egypt; 5 Department of Pharmacology and Toxicology, Faculty of Pharmacy, Al-Azhar University, Cairo, Egypt; 6 Preclinical & Translational Research Center, Faculty of Pharmacy, Ain Shams University, Cairo, Egypt

**Keywords:** Breast cancer, Tamoxifen, Hypoxia, Metabolic alterations, Mitochondrial activity

## Abstract

Only a few investigations, to our knowledge, have examined the bioenergetics of Tamoxifen (TMX) resistant individuals and reported altered mitochondrial activity and metabolic profile. The primary cause of TMX resistance is firmly suggested to be metabolic changes. Metabolic variations and hypoxia have also been linked in a bidirectional manner. Increased hypoxic levels correlate with early recurrence and proliferation and have a negative therapeutic impact on breast cancer (BC) patients. Hypoxia, carcinogenesis, and patient death are all correlated, resulting in more aggressive traits, a higher chance of metastasis, and TMX resistance. Consequently, we sought to investigate the possible role of the metabolic/hypoxial axis Long non-coding RNA (LncRNA) Taurine up-regulated 1 (TUG-1), Micro-RNA 186-5p (miR-186), Sirtuin-3 (SIRT3), Peroxisome Proliferator Activator Receptor alpha (PPAR-α), and Hypoxia-Inducible Factor-1 (HIF-1) in the development of TMX resistance in BC patients and to correlate this axis with tumor progression. Interestingly, this will be the first time to explore epigenetic regulation of this axis in BC.

## Tamoxifen treatment in breast cancer

Tamoxifen (TMX), a triphenylethylene derivative, was the predominant hormonal-based treatment for both adjuvant and metastatic BC. It is still one of the most effective treatments for extending both recurrence-free and overall survival [1]. Among the several anticancer medications used today, TMX merits special consideration. TMX was first classified as an anti-estrogen, because it inhibits estrogen receptors in breast tissue, limiting the effects of estrogen. TMX, on the other hand, operates as an agonist for estrogen receptor (ER) in certain body locations such as the endometrium, liver, and bone [2]; as a result, it was classed as a selective estrogen receptor modulator (SERM). This drug has been widely used to treat BC and as a preventative treatment in women at high risk of getting the condition [3].

ER-positive cases account for almost two-thirds of all BC cases. Because the receptor promotes mammary epithelial cell proliferation, it is an important target in anti-hormonal cancer treatment. TMX, which has assisted millions of women since its discovery 50 years ago, is one of the most often recommended ER antagonists for first-line treatment. TMX is known to have direct and indirect impacts on cellular lipid metabolism in addition to its primary anti-cancer actions. It has been demonstrated to lower blood cholesterol levels and protect against cardiovascular disease [4, 5]. Table 1 clearly summarizes the main pros and cons of TMX.

**Table 1:** Tamoxifen pros and cons

Tamoxifen pros	Tamoxifen cons
Decreases recurrence	40% of patients eventually relapse
Reduces cell proliferation	Development of recurrent tumors
Prevents the development of cancer	Resistance develops over time
Induces apoptosis	
Reduces the risk of developing invasive breast cancer	

Treatment with SERMs, particularly TMX, has reduced BC mortality by 25–30%. Approximately 30% of women treated with TMX face recurrence in the next decade due to the development of late resistance after continuous exposure to the medication, particularly in the metastatic situation [6]. It is suggested that BC cells can develop TMX resistance by deregulation of several cellular pathways, depending on their genetic profile. Different mechanisms of TMX resistance in BC include alterations in ER, ER signaling cascade, metabolic alterations, and mitochondrial bioenergetics [7–9]. Several molecular processes, often involving the replacement of the pro-proliferative ER signaling by other signaling pathways like EGFR/HER2 or IGFR, have been hypothesized as the cause of TMX resistance. The participation of ER36, a structurally distinct isoform of ER that is enhanced in TMX-resistant cells, is suggested by other data. On the other hand, it has been proposed that breast tumors expressing low amounts of another ER isoform (ER) may be resistant to TMX therapy. Additionally, several research indicate that TMX-resistant cells have dysregulated micro-RNA expression profiles.

Indeed, several micro-RNAs show direct modulation of the ER pathway (miR18a, miR-18b, miR-22, miR-193b, miR- 206, miR-221/222, miR-301a, and miR-302c), linking them with the acquisition of resistance to TMX treatment. Additionally, data proposing involvement of p130Cas/Src signaling as well as NFκB pathway in the resistance has been published [8]. Tamoxifen metabolism varies between individuals, which may affect therapeutic efficacy and the levels of metabolites in the serum [10]. Tamoxifen also downregulates hypoxia-regulated genes.

## Association of metabolic/mitochondrial/hypoxial axis with TMX function

**Fig. 1:**
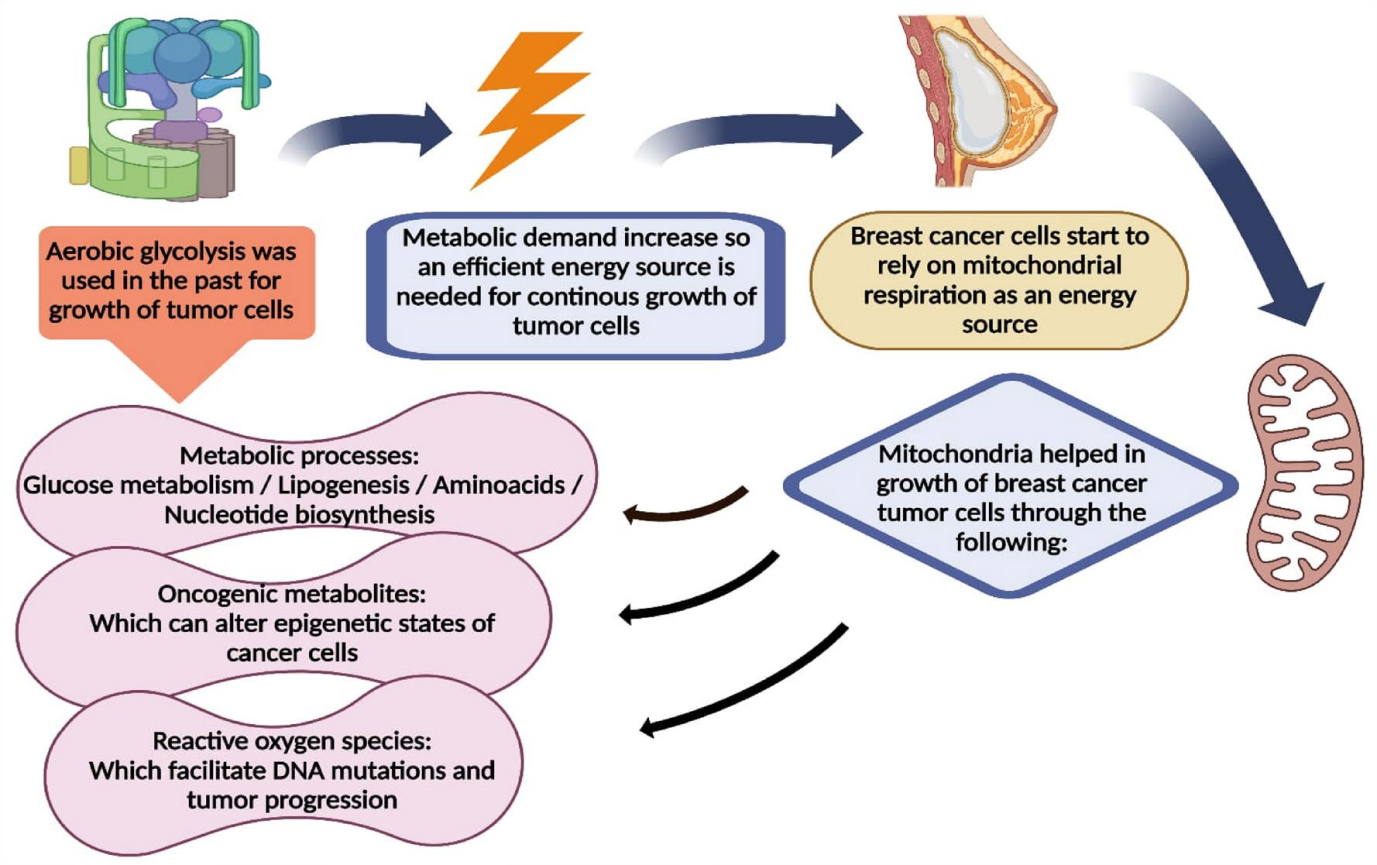
Schematic diagram of mitochondrial metabolic function in breast cancer tumor cells

Recently, incorporated mitochondrial bioenergetics and metabolic alterations are strongly introduced as one of the major factors of TMX resistance [8]. The biology of rapidly growing tumors often results in increasing metabolic demand, necessitating the utilization of a more effective energy source for continuous growth. New cancer studies demonstrate that mitochondrial respiration still plays a substantial role in carcinogenesis despite the fact that aerobic glycolysis has long been recognized as a crucial characteristic of cancer cells [11]. For instance, various tumor cells including BC cells have been shown to rely on mitochondrial respiration [12]. Furthermore, mitochondrial metabolic pathways or activities, such as glucose metabolism, lipogenesis, amino acid metabolism, and nucleotide biosynthesis, are thought to contribute to tumor growth [13]. Mitochondria produce carcinogenic metabolites, which can change cancer cells' epigenetic states. Furthermore, mitochondria produce reactive oxygen species (ROS), which promote DNA alterations and tumor growth [14]. Figure 1 shows a schematic diagram summarizing the mitochondrial metabolic function in BC tumor cells.

**Fig. 2:**
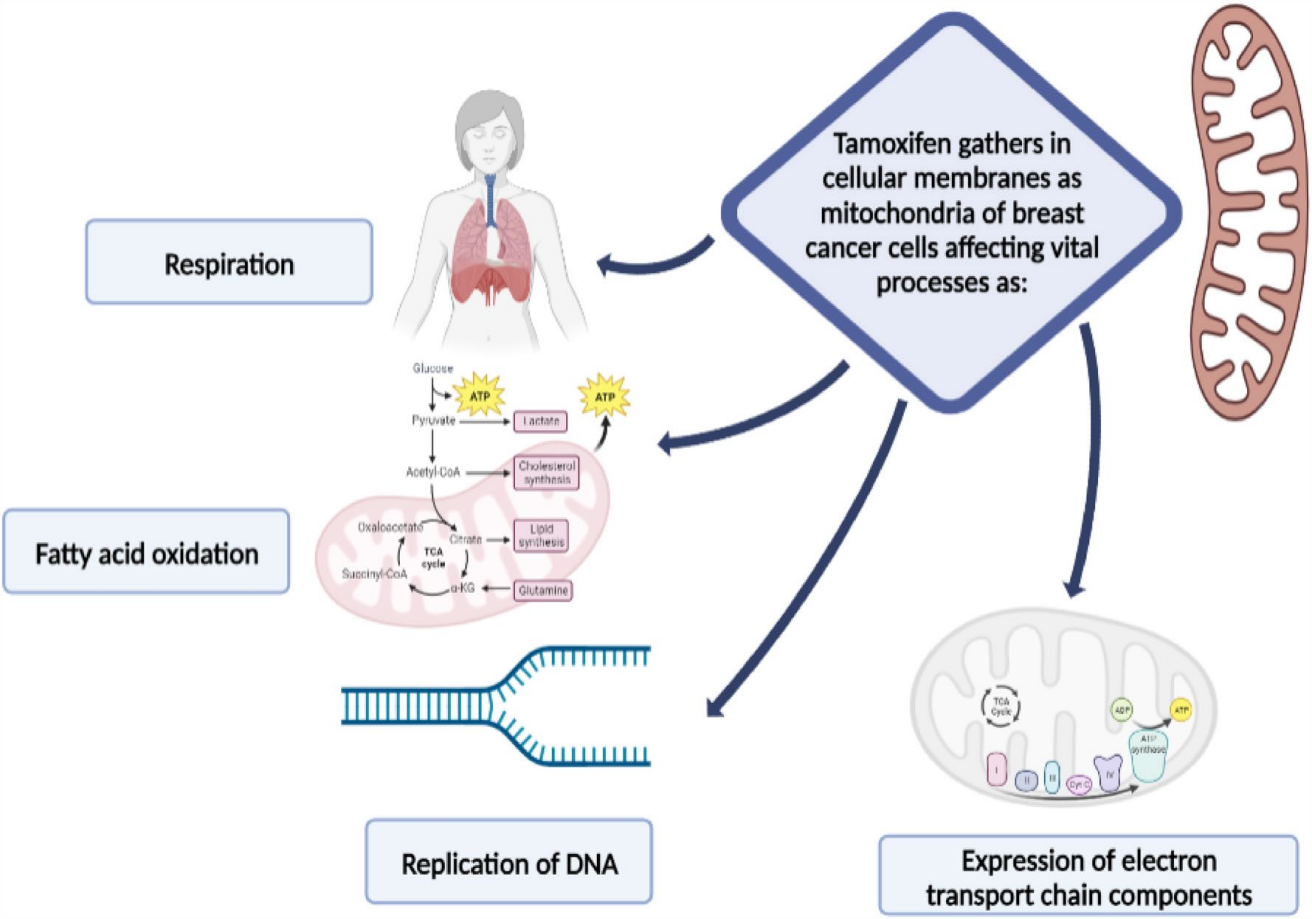
Schematic diagram showing the effect of tamoxifen on mitochondrial/metabolic function in breast cancer tumor cells

The function of the mitochondria can be directly impacted by TMX. It preferentially gathers in cellular membranes, and its accumulation within mitochondria affects vital processes like respiration, fatty acid oxidation (FAO), production and replication of mitochondrial DNA, and expression of mitochondrially encoded components of the electron transport chain. Figure 2 shows a schematic diagram summarizing the effect of TMX on mitochondrial/ metabolic function in BC tumor cells [15].

Knowing that one of the primary causes of TMX resistance is metabolic dysregulations and hypoxia involvements [8], since it is still not entirely apparent, investigating the mechanism of TMX resistance in BC patients is crucial.

Additionally, there is a gap in the market for a trustworthy biomarker for TMX resistance in BC patients. It is proposed that the BC cells can acquire TMX resistance by dysregulations of different cellular pathways, dependent on their individual molecular phenotypes**.**

**Fig. 3:**
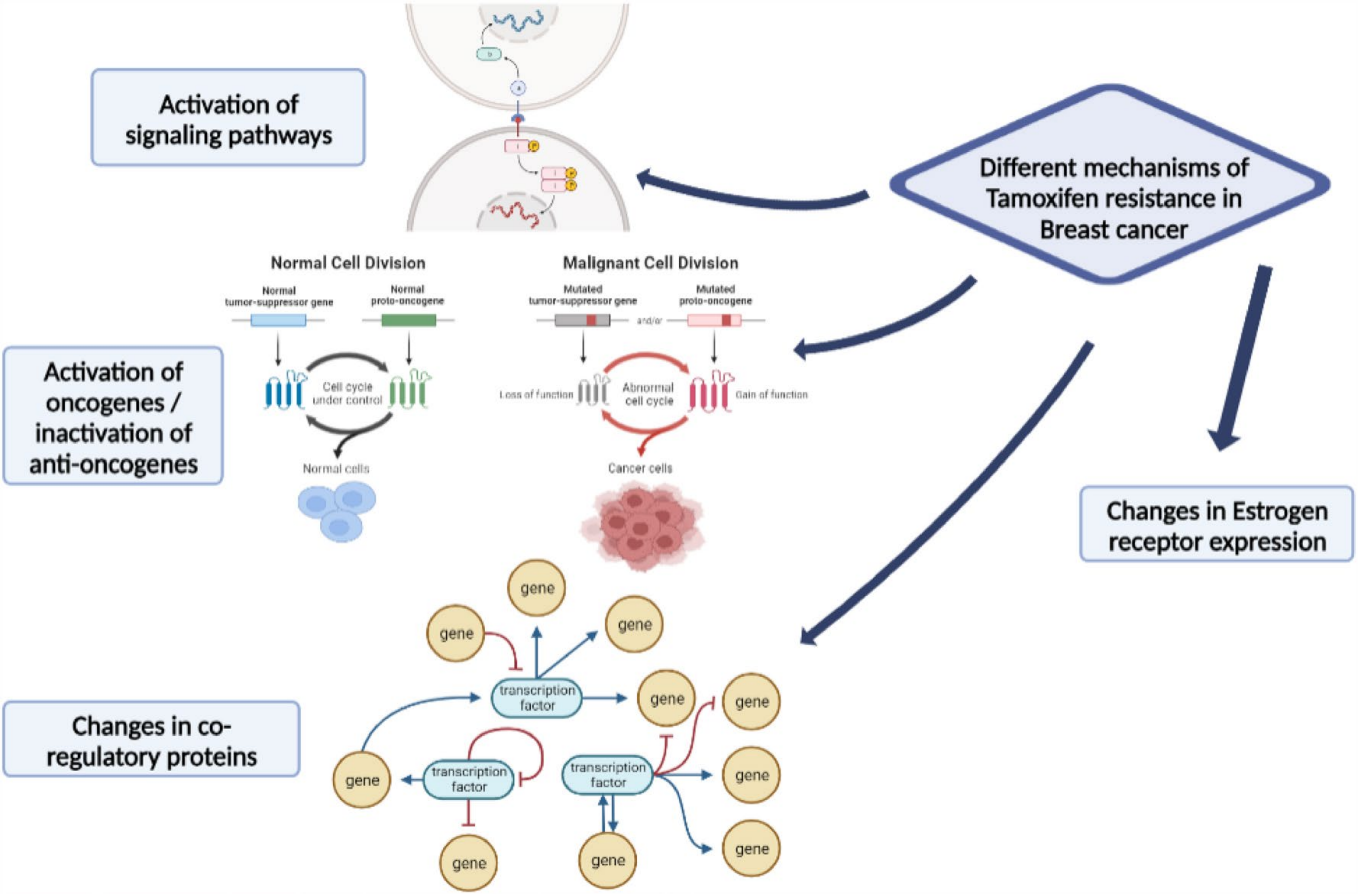
Schematic diagram summarizing mechanisms of tamoxifen resistance in breast cancer

Modifications to TMX’s direct targets as well as the activation of alternate signaling pathways are examples of resistance mechanisms. Differential gene expression and pathway analysis demonstrated that, depending upon the cell type, TMX resistance is not caused by a single common mechanism but instead includes a number of functional pathways. The activation of oncogenes, the inactivation of anti-oncogenes, changes in ER expression, changes in co-regulatory proteins, and the involvement of growth factor signal pathways are only a few of the components that are connected to TMX resistance mechanisms. Figure 3 shows a schematic diagram summarizing mechanisms of TMX resistance in BC [16, 17].

**Fig. 4:**
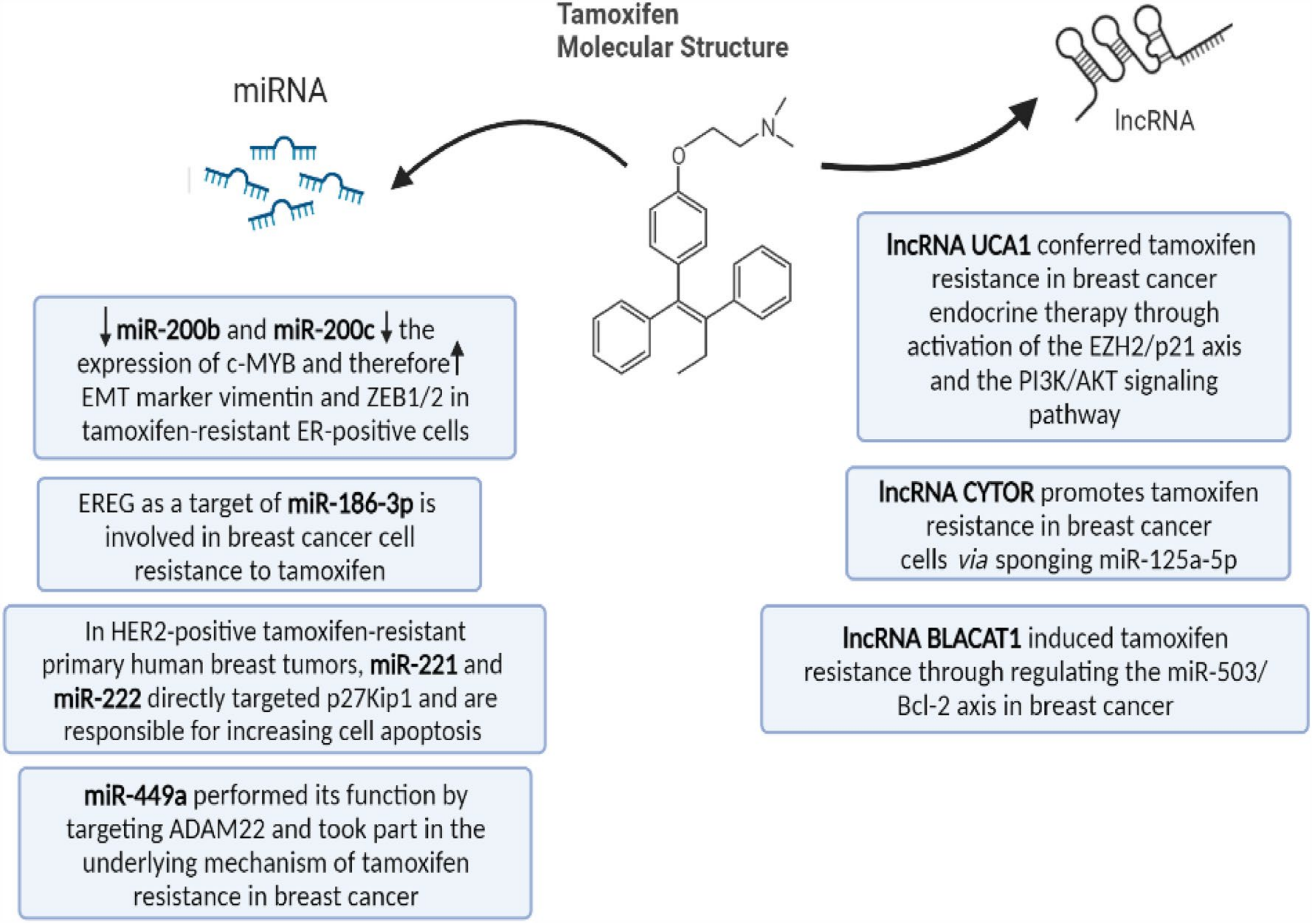
Schematic diagram summarizing the effect of different non-coding RNAs on tamoxifen resistance

Non-coding RNAs' capacity to regulate gene expression makes them potential targets or important regulators of the tumor TMX resistance. The activation or deregulation of several pathways involved in the emergence of TMX resistance is frequently caused by the control of gene expression by micro-RNAs. Figure 4 shows a schematic diagram summarizing the effect of different non-coding RNAs on TMX resistance [18].

Moreover, dysregulations in hypoxia-related genes can lead in turn to TMX resistance. The effect of different hypoxic genes on TMX resistance was previously illustrated in several trials. A clinical trial of 187 patients with BC found that overall response to TMX treatment decreased with increased tumor Hypoxia-Inducible Factor alpha (HIF-1) [19]. Furthermore, a previous study revealed that increased HIF-1 expression was associated with TMX resistance [20].

HIF-1 can lead to BC resistance to endocrine drugs as TMX and cytotoxic drugs through upregulation of autophagy [21]. TMX also downregulates hypoxia‐regulated genes and increases vascularization in PDAC tissues [22]. When ERα^+^ BC cells were transduced with HIF-1, the cancer cells became much more resistant to TMX [23].

Accordingly, we chose this particular mitochondrial/metabolic axis as a novel pathway to address TMX resistance in BC patients from the epigenetic state to the protein state of cancer cells.

### Taurine up-regulated 1

Long non-coding RNA (LncRNA) has been shown to have a key role in the genesis, progression, and anti-estrogen resistance in BC. Furthermore, a novel molecular categorization of BC has been proposed based on LncRNA expression, and nearly two-thirds of the LncRNAs expressed in BC were shown to be localized in enhancer areas [3].

Recent research has found that nuclear-encoded LncRNAs affect mitochondrial dynamics. LncRNAs can govern cell metabolism and cancer cell survival by influencing mitochondrial components such as complexes I–IV and other subunits such as ATPase on the mitochondrial inner membrane. In addition to gene transcription, LncRNAs can direct protein translation to affect cancer metabolism and mitochondrial function. So far, LncRNAs have been investigated as possible cancer molecular biomarkers, contributors to treatment resistance and disease progression, and prospective therapeutic targets along the metabolic route [24].

A 7,598-nucleotide LncRNA sequence known as Taurine up-regulated 1 (TUG-1) was detected on chromosome 22q12.2 during a genomic search of taurine-treated mouse retinal cells. TUG-1 is substantially associated with growing tumor size, advanced clinical stage, and distant metastasis. It has been shown that TUG-1 is elevated in BC [25]. Recent research suggests that TUG-1 regulates genes through a number of different methods, notably by acting as a micro-RNA sponge [26, 27]. It was also poorly controlled in the carcinogenesis process as either an oncogene or a potential tumor inhibitor [28].

Targeting micro-RNAs, TUG-1 works as a competitive endogenous RNA to block their biological actions [29]. This leads to changes in the expression level of downstream target genes [30]. TUG-1 may alter gene expression by distinct mechanisms controlling different biological processes. These processes include but are not limited to the following [31];

1. Cell migration, invasion, differentiation, and death.
2. Resistance to drugs and radiation.
3. Angiogenesis
4. Mitochondrial bioenergetics.
5. Epithelial-mesenchymal transition.
6. The control of blood–tumor barrier permeability.

The curative effect of conventional carcinogenic drugs like TMX is established by TUG-1’s high tendency for their severe adverse effects. Inventive research that compared the plasma levels of TUG-1 in the two groups discovered that patients who were resistant to TMX had substantially greater TUG-1 levels than people who were TMX responsive [28]. TUG-1 downregulation can be used as a distinguishing feature of cancer therapy, because TMX medication was shown to reduce TUG-1 expression [32]. The results of recent studies supported the roles of TUG-1 in myocardial infarction, possibly through mitochondrial dysfunction and pyroptosis mediated by increased ROS generation [33].

### Micro-RNA 186-5p

Several micro-RNAs with oncogenic potential have been discovered in BC. Oncogenic micro-RNAs demonstrate their oncogenic potential by increasing cell proliferation, cancer, and/or metastasis, as well as boosting angiogenesis [34]. Micro-RNAs are unregulated in cancer and can function as tumor suppressors, inhibiting tumor development, or as oncogenes (termed oncomiRs), which are overexpressed in cancer and promote tumor formation. Micro-RNAs have been proposed as crucial prognosticator indicators in BC, and several current investigations are attempting to uncover micro-RNAs with the capacity to predict TMX response [3].

Overall, micro-RNA-based treatments have demonstrated significant therapeutic potential for cancer and infectious disorders. So far, various methods based on micro-RNA mimics or micro-RNA inhibitors have entered clinical trials. The human Saos-2 cell line was the site of the 2003 discovery of Micro-RNA 186-5p (miR-186). It is crucial to understand the mechanisms underlying the conflicting findings on miR-186's role in cancers, since doing so might delay the usage of this target for diagnostic and therapeutic purposes [35]. miR-186 reverses TMX resistance in BC by increasing cell death and decreasing cell proliferation. It should be noted that glycolysis is adversely regulated in BC that is resistant to TMX by miR-186-mediated reduction of epiregulin [36]. Through genomic and nongenomic/membrane-initiated pathways, estrogens and other ER ligands like TMX and endocrine disruptors control a variety of physiological consequences that change the cellular expression of micro-RNAs. There have been reports of micro-RNA alterations in fish, mice, rats, and human BC cells in response to TMX; however, the precise processes underlying these reactions have received very little attention [37].

Moreover, miR‐186 may also affect the Hypoxia-Inducible Factor alpha (HIF-1)-dependent lung structure maintenance program [38]. Micro-RNAs may play a key role in the development of tumors and the spread of cancer, according to research showing their participation in the control of vital cellular homeostasis pathways. It is becoming more and more obvious that metabolic re-programming is important for tumor development and metastasis [39]. Strong evidence points to the crucial role that micro-RNAs play in energy metabolism, namely in the lipid and glucose metabolism as well as the production of amino acids. Additionally, micro-RNAs have the ability to recognize and alter metabolic elements at the transcriptional level, which is crucial for both non-cancerous and cancerous cells [40]. Changes in mitochondrial micro-RNAs, a key regulator of mitochondrial functioning, have been discovered in several pathologies, including BC. Complex processes govern how mitochondrial micro-RNAs affect mitochondrial activity in cancer [41].

### Sirtuin-3

Sirtuins are mitochondrial proteins that regulate the function of several mitochondrial metabolic proteins. Sirtuin-3 (SIRT3) specifically deacetylates and controls the activities of many proteins involved in mitochondrial biogenesis, ROS homeostasis, and metabolic pathways in mitochondria [42].

Previous research has shown that SIRT3 regulates the mitochondrial adenosine tri-phosphate (ATP) synthesis machinery via effects on the respiratory chain, implying that SIRT3 may be a critical mediator of energy required under a variety of stress circumstances. Essentially, SIRT3 may govern ATP generation, at least in the heart and muscle, via regulating AMP-activated protein kinase (AMPK), a sensor of cellular energy status. Once activated, AMPK promotes catabolic pathways, primarily through increasing oxidative metabolism and mitochondrial biogenesis to create ATP, while suppressing anabolic pathways that entail ATP consumption [43]. Recently, SIRT3’s role as a mitochondrial localized tumor suppressor was identified. It is crucially demonstrated that SIRT3 overexpression is sufficient to prevent HIF-1 stabilization under hypoxia and to suppress carcinogenesis, revealing a unique role for SIRT3 in the upkeep and development of cancer [44]. SIRT3 is now identified as a human-related protein that regulates cellular energy metabolism at both the transcriptional (nucleus) and post-transcriptional (mitochondria) levels. SIRT3 appears to play a dual role in cancer cells, acting as both a tumor suppressor and a promoter. Indeed, it is downregulated in many malignancies, including prostate, hepatocellular, and breast carcinomas, but overexpressed in head and neck squamous carcinoma, where it regulates ROS to a level capable of blocking apoptosis and ensuring an aggressive and proliferative tumor phenotype [45]. SIRT3 might be considered as a potential target for overcoming TMX resistance in treatment of breast cancer [46].

### Peroxisome proliferator activator receptor alpha

Peroxisome proliferator-activated receptors’ proteins belong to superfamily of proteins termed nuclear hormone factors [47]. The family of Peroxisome Proliferator Activator Receptors is represented by the following three members: Peroxisome Proliferator Activator Receptor alpha (PPAR-α), Peroxisome Proliferator Activator Receptor -δ, and Peroxisome Proliferator Activator Receptor -γ. They play an essential role in energy metabolism; however, they differ in the spectrum of their activity [48].

It has been demonstrated that PPAR-α regulates glucose metabolism, lipoprotein metabolism, liver inflammation, amino acid metabolism, and hepatocyte proliferation. Synthetic Peroxisome Proliferator Activator Receptor agonists reduce plasma triglycerides while increasing plasma high-density lipoprotein levels, and are consequently used therapeutically to treat dyslipidemia [49].

Depending upon the type of ligand or tissue of origin, activation of PPAR-α either potentiates or attenuates tumor progression [50]. Extensive PPAR-α activation has been linked to tumor development progression in a variety of malignancies, including triple-negative BC. As a result, this route may be important in carcinogenesis, particularly in BC [51].

Peroxisome Proliferator Activator Receptor modulators, which include agonists and antagonists, may provide a unique technique for preventing and treating a variety of cancers. As a result, they are linked to cancer cell proliferation, differentiation, and death, lending credence to the anticancer potential of Peroxisome Proliferator Activator Receptor modulators. In terms of Peroxisome Proliferator Activator Receptor agonists, they play a vital role in the prevention of several malignancies, including BC [52]. PPAR-α agonists reduced hypoxia-induced HIF-1 expression and activity in cancer cells before hypoxia, and the addition of a PPAR-α antagonist lessened the suppression of HIF-1 signaling [53].

PPAR-α is highly expressed in metabolically active tissues, such as liver, heart, skeletal muscle, intestinal mucosa, and brown adipose tissue [54]. In vivo and In vitro studies demonstrate that PPAR-α plays a central role in lipid and lipoprotein metabolism, and thereby decreases dyslipidemia associated with metabolic syndrome [55]. The tricarboxylic acid cycle and oxidative phosphorylation were two genes whose expression was lowered by overexpression of PPAR-α, which also caused mitochondria to become disorganized, modify their cristae density and architecture. These findings imply that altered mitochondrial structure and metabolic function are associated with aberrant PPAR-α expression [56].

### Hypoxia-inducible factor alpha

The major effector of the hypoxia pathway is Hypoxia-Inducible Factor, a heterodimer protein consisting of a stable hypoxia-inducible factor-1β sub-unit along with HIF-1, which undergoes ubiquitin-mediated degradation under normal oxygen levels. Upon stabilization, HIF-1 forms a complex with Hypoxia-inducible factor-1β, which along with cofactors like CBP and p300 activate the transcription of several genes involved in processes that are vital for the survival and spread of tumor cells to metastatic sites [57].

HIF-1 is the functional sub-unit that is degraded by proteasomes under normoxia, which stabilizes and accumulates under hypoxia [58]. It has been shown that the activation of the hypoxia pathway is crucial for the development of angiogenesis, extracellular matrix remodeling, the formation of a pre-metastatic pool, invasion, and extravasation at the metastatic site in BC as well as other solid tumors. In addition to affecting the alternative splicing of pre-micro-RNA transcripts, hypoxia has been shown to affect the transcriptional activity of genes controlled by HIF-1. Numerous investigations revealed a connection between hypoxia and cancer development, metastasis, unsuccessful therapy, and patient death [59, 60].

The TMX resistance of hypoxic BC cells was demonstrated, and TMX sensitivity was recovered by inhibiting HIF-1. Epithelial growth factor receptor, which is related to TMX resistance, was discovered to interact with HIF-1 to cause TMX resistance [20].

By increasing the expression of metabolic enzymes, HIF-1 modifies energy metabolism. As a result, it is thought to be the primary force behind metabolic adaptation to hypoxia. These HIF1-dependent genes regulate how much glucose is used by cells and cut down on oxygen consumption to lessen the need for oxygen during metabolism while keeping proper ATP levels. Additionally, by boosting mitophagy and reducing mitochondrial biogenesis, HIF-1 can lower the bulk of the mitochondria. Therefore, at times of low oxygen supply, HIF1 plays a key role in regulating cellular metabolic strategy. It is interesting to note that certain HIF-1 hydroxylases can control metabolic processes through different ways [61].

## Conclusion

Evidence is mounting that non-coding RNAs are essential for the epigenetic control of target genes. Consequently, they are considered potential therapeutic targets and diagnostic indicators in BC [62]. Indeed, a number of LncRNAs and micro-RNAs exhibit direct regulation of anomalies in mitochondria and metabolism [63]. Intriguingly, study on LncRNAs, micro-RNAs, and TMX resistance in BC has advanced recently, highlighting the novel function of LncRNAs and micro-RNAs in endocrine therapy in BC [64, 65]. In BC patients, it is critical to search for TMX resistance mechanisms. Additionally, it is critical to look for a marker that can effectively manage BC patients who are TMX-resistant.

Examining the remarkable effects that TMX resistance has on BC patients is vital to comprehend, observe, and predict disease processes. The past 10 years have seen an increase in knowledge regarding the function of LncRNAs in cancer. Outstanding efforts have aided in the identification of potent drugs and potential targets for the therapy of BC patients.

LncRNAs are good predictive markers for BC and also function as oncogenes and tumor suppressors [62]. The identification of TUG-1 as an important regulator of BC development suggested that it might function as an indicator for the detection and management of BC [66]. Future studies on TUG-1 indexing mechanisms in cancer cells could lead to a variety of inventive medicinal strategies for the management of tumors. The focus of present and future studies must be on comprehending the fundamental molecular mechanisms of TUG-1 [31]. TUG-1 has also been discovered to be up-regulated in cardiac cells that are oxygen-deprived [67].

Growing data demonstrated that micro-RNAs play critical roles in both the formation of tumors and the progression of malignancy, and that micro-RNA-based novel anticancer treatments are currently being developed [68]. Earlier research showed that miR-186 was increased in a number of cancers, where it increased cell growth and motility while blocking a number of targets to prevent apoptosis [35]. Recent research suggests that SIRT3 is a novel factor in BC cells' TMX resilience [8]. SIRT3 promotes drug resistance in TMX-resistant BC cells [69].

In a similar vein, numerous Sirtuins have been shown to influence HIF-1 function; SIRT3 specifically destabilizes it. In several tumor forms, inhibiting the HIF-1 enzyme is a potential therapeutic target [70]. Hypoxia has been demonstrated to affect the response to TMX in numerous BC cell lines and to down-regulate ER in ER-positive BC patients [71].

**Fig. 5:**
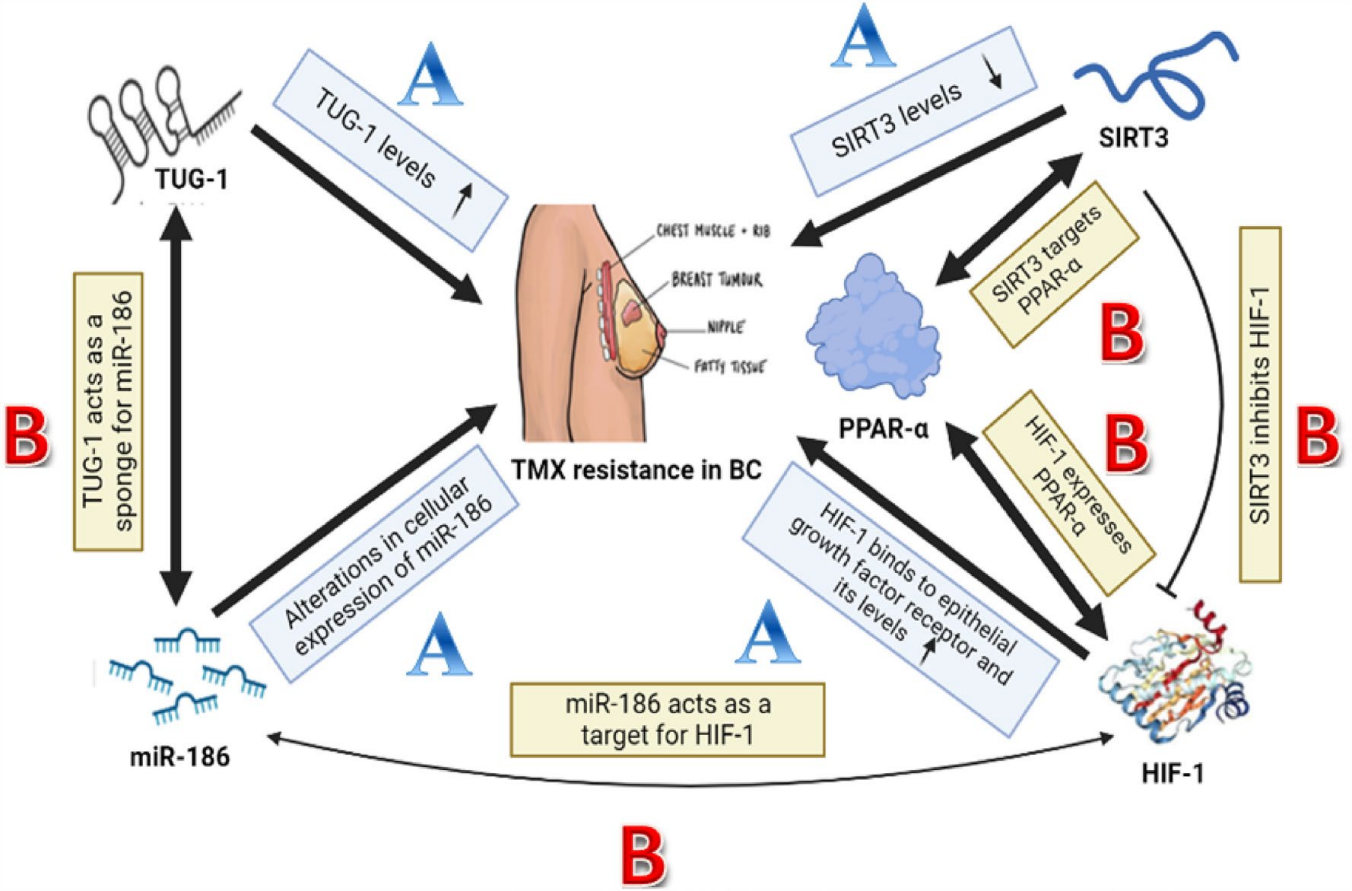
Possible contributions to TMX resistance by the novel mitochondrial metabolic axis

To reduce fatalities and improve quality of life, research into early diagnostic markers is continuing, and non-invasive methods of forecasting TMX resistance are of critical importance. As a result, the objective of this study was to research and examine any potential contributions to TMX resistance in BC patients made by the novel mitochondrial and metabolic axis TUG-1, miR-186, SIRT3, PPAR-α, and HIF-1. These various contributions and correlations are clearly summarized in Fig. 5. This could aid in a greater comprehension of the crucial function that altered mitochondrial and metabolic pathways play in TMX resistance.

As shown in Fig. 5, *letter A shows all contributions between TMX and our axis as follows; i*nventive research that compared the plasma levels of TUG-1 in the two groups discovered that patients who were resistant to TMX had substantially greater TUG-1 levels than people who were TMX responsive [28]. Thus, high TUG-1 levels lead to TMX resistance in BC. Through genomic and nongenomic/membrane-initiated pathways, estrogens and other ER ligands like TMX and endocrine disruptors control a variety of physiological consequences that change the cellular expression of micro-RNAs. There have been reports of miR-186 alterations in fish, mice, rats, and human BC cells in response to TMX [37]. SIRT3 might be considered as a potential target for overcoming TMX resistance in treatment of breast cancer [46]. Thus, low SIRT3 levels lead to TMX resistance in BC. Epithelial growth factor receptor, which is related to TMX resistance, was discovered to interact with HIF-1 to cause TMX resistance [20]. Thus, high HIF-1 levels lead to TMX resistance in BC.

On the other hand, *letter B shows all correlations between our axis components as follows;*

Recently, studies showed TUG-1 to function as a sponge for miR-186 [72, 73]. SIRT3 overexpression is sufficient to prevent HIF-1 stabilization under hypoxia and to suppress carcinogenesis [44]. Earlier cancer studies indicated that miR-186 may target HIF-1 [74]. SIRT3 was a direct, positively regulated target of PPAR-α [75]. To sustain PPAR-α translation, HIF-1 may additionally collaborate with other hypoxia-modified transcriptional regulators [76].

Accordingly, future large-scale and experimental studies are required to unravel the molecular mechanism underlying the clinical actions of TMX in BC. Future studies should be aimed to identifying the role of TUG-1 and miR-186 within the confines of well-known tumor suppressor and metabolic pathways. Last but not least, physicians may start to use micro-RNAs and LncRNAs as new non-invasive biomarkers instead of traditional ones for early detection of BC.

## Data Availability

All data are included in the manuscript.

## Abbreviations

AMPK: AMP-activated protein kinase
ATP: Adenosine tri-phosphate
BC: Breast cancer
ER: Estrogen receptor
FAO: Fatty acid oxidation
HER2: Human Epidermal growth factor Receptor-2
HIF-1: Hypoxia-Inducible Factor alpha
LncRNA: Long non-coding RNA
miR-186: Micro-RNA 186-5p
PPAR-α: Peroxisome Proliferator Activator Receptor alpha
ROS: Reactive oxygen species
SERM: Selective estrogen receptor modulator
SIRT3: Sirtuin-3
TMX: Tamoxifen
TUG-1: Taurine up-regulated 1
